# ITERL: A Wireless Adaptive System for Efficient Road Lighting

**DOI:** 10.3390/s19235101

**Published:** 2019-11-21

**Authors:** María García-Castellano, Juan Manuel González-Romo, Juan Antonio Gómez-Galán, Juan Pablo García-Martín, Antonio Torralba, Ventura Pérez-Mira

**Affiliations:** 1 Departamento de Ingeniería Electrónica, ETS Ingenieros, Universidad de Sevilla, 41092 Seville, Spain; mgarcia@gie.us.es (M.G.-C.); jgonzalez@gie.us.es (J.M.G.-R.); jpgarcia@gie.us.es (J.P.G.-M.); torralba@us.es (A.T.); 2 Departamento de Ingeniería Electrónica, Sistemas Informáticos y Automática, Universidad de Huelva, 21007 Huelva, Spain; 3 Departamento de Ingeniería Química y Ambiental, ETS Ingenieros, Universidad de Sevilla, 41092 Seville, Spain; vperez4@us.es

**Keywords:** IoT, wireless sensors, low-power, smart lighting, road lighting, intelligent transportation systems, traffic intersections

## Abstract

This work presents the development and construction of an adaptive street lighting system that improves safety at intersections, which is the result of applying low-power Internet of Things (IoT) techniques to intelligent transportation systems. A set of wireless sensor nodes using the Institute of Electrical and Electronics Engineers (IEEE) 802.15.4 standard with additional internet protocol (IP) connectivity measures both ambient conditions and vehicle transit. These measurements are sent to a coordinator node that collects and passes them to a local controller, which then makes decisions leading to the streetlight being turned on and its illumination level controlled. Streetlights are autonomous, powered by photovoltaic energy, and wirelessly connected, achieving a high degree of energy efficiency. Relevant data are also sent to the highway conservation center, allowing it to maintain up-to-date information for the system, enabling preventive maintenance.

## 1. Introduction

Until recently, communication was considered to be a type of relationship among humans. The Internet was created in the 1970s to exchange information among connected computers. The capability of connecting smart devices, identified with a unique address, to the Internet paved the way for the so-called Internet of Things (IoT). It is expected that "things" communicating with other "things" on behalf of people will dominate the future of Internet communications. In fact, it is estimated that, at some time between 2008 and 2009, the number of devices connected to the Internet became higher than that of connected people. Since then, this number has grown exponentially.

The integration of the IoT with intelligent traffic systems has recently been explored [1,2,3,4,5]. The IoT can provide both infrastructure and vehicles with sensors that can measure general traffic conditions, ambient variables, vehicle variables (such as position and speed), and driver conditions. For example, with this information it is possible to measure the intensity of traffic and the degree of traffic congestion, as well as to detect risk situations. Through local and global smart processing, it is possible to act on traffic control systems and interact with the drivers in order to reduce waiting times, increase the efficiency of transport systems, and decrease the accident rate. Ultimately, from the information obtained from connected devices, it is possible to develop decision-making tools and to carry out medium- and long-term planning of investments in infrastructure and control systems. 

Road lighting represents one of the most efficient measures that an intelligent transport system can take to reduce accidents. The effect of introducing or improving lighting on the number of road accidents has been extensively studied. The authors in [6,7] found a statistically significant dose-response relationship between average road luminance and road safety. Although there are differences in the literature regarding the percentage of reduction in the night/day crash ratio due to the presence of lighting, the common conclusion is that this is not a small number, even though it is not as high as the 30% reported in some studies conducted at the start of this century [8,9,10]. A 19% reduction in the night/day crash ratio due to the presence of destination lighting at stop-controlled cross-intersections was reported in [11]. Authors in [12,13] reduced this figure down to 12% when other safety influencing features were taken into account. 

It is well known that a high percentage of road fatalities (up to 55% in Europe in 2017) occur in rural roads [14,15], due to the fact that they are highly sensitive to ambient conditions, among other reasons. Nonetheless, many secondary and rural road intersections are not currently lit, due to problems with accessing the power grid or to the high operational and maintenance costs of lighting systems. The lighting of intersections based on photovoltaic energy has turned out to be expensive due to the high costs of the batteries needed to run the lights overnight and their associated power equipment. 

Significant savings could be achieved by using recent advances in information and communication technologies (ICTs). For example, an object classification method for common traffic scenarios using light detection and ranging (LIDAR) sensors is characterized in [16]. However, the application of ICTs to road safety remains low, especially when compared to other sectors with great economic or social impact. This scenario is changing due to three factors: the emergence of power-efficient lamps based on LED technology; the decreasing cost and increasing efficiency of power equipment; and the use of adaptive lighting systems that turn streetlights on only when necessary, reducing the size and cost of the batteries. Their combined effect not only enables the design of autonomous photovoltaic-powered LED luminaires, but also reduces their installation, operation, and maintenance costs, allowing streetlights to be deployed in places where it was previously not possible, or providing more light in areas that were previously dimly lit.

This paper describes the results of the IoT technologies for efficient road lighting (ITERL) project, which combines the use of LED luminaires with a smart system based on power-efficient IoT techniques that control the on–off function and light intensity of the streetlights, according to road, traffic, and ambient conditions. For this purpose, autonomous streetlights have been developed with wireless IP connectivity, specially designed to illuminate remote intersections with little traffic. The streetlights are switched on when incoming vehicles are detected by means of autonomous sensors that are also wirelessly connected; then, they are gradually turned off when the vehicles leave the intersection. Energy consumption is optimized, since the streetlights are off during the day, except when visibility conditions are not adequate.

## 2. Preliminary Considerations

**Figure 1:**
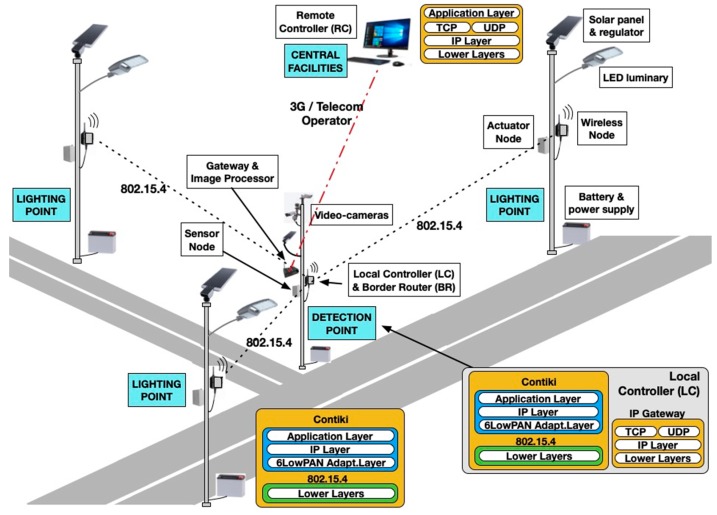
General architecture of Internet of Things (IoT) technologies for efficient road lighting (ITERL).

The development of a smart lighting system must address knowledge related to various technological fields: sensors, wireless communication, streetlights, renewable generation, and energy control and storage. To consolidate the diverse requirements of all these fields, the general architecture of Figure 1 was selected for ITERL. To build such a system, it is necessary to develop:

- A power-efficient, low cost lighting system using:
  ○ Power-efficient streetlights based on LED diodes;
  ○ Smart streetlight controllers with high-energy efficiency and dimming control;
  ○ High-performance electronic converters for the energy conversion required to power the streetlights and the control systems;
  ○ Advanced energy storage systems, with high capacity and easy maintenance;
  ○ Microcontroller-based controllers connected through a cellular modem;
  ○ Sensors for monitoring ambient variables, as well as for the detection of vehicles;
  ○ Autonomous wireless communication among system components.
- Safe algorithms for lighting controls in intersections, including:
  ○ Regulation of the illumination level depending on ambient variables, vehicle detection, and charge level of energy storage elements;
  ○ Robust and redundant techniques that guarantee safe and reliable operation.
- A communication link with the central operation and management center, using a cellular network to get updated information about the state of the energy, control, and lighting elements.
- A maintenance system able to provide:
  ○ Early detection of malfunction or aging of the lighting, control, storage, and energy conversion components, in order to enable their preventive maintenance;
  ○ Rapid detection of fault, emergency, and alarm situations, which are immediately reported to the central station.

## 3. Hardware Description

**Figure 2:**
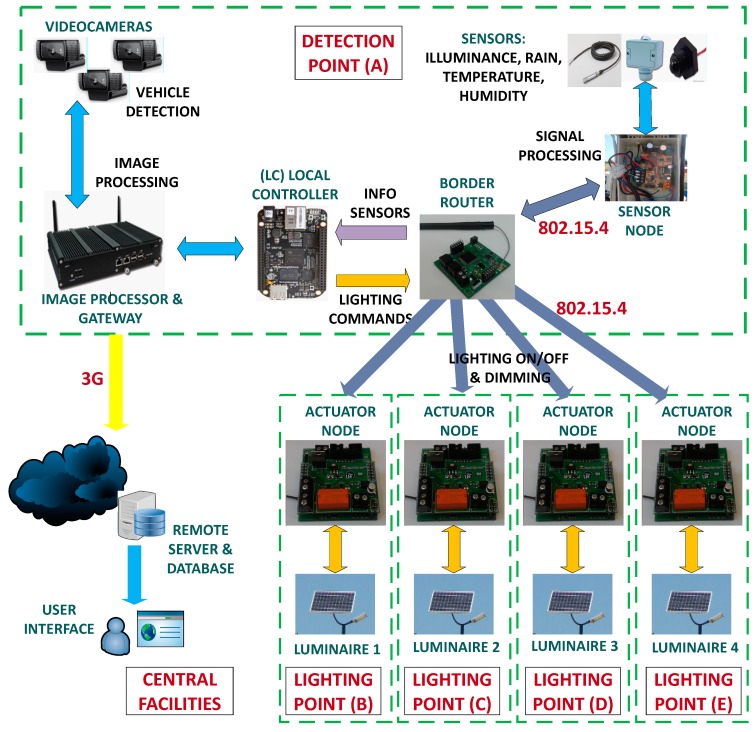
Functional diagram of ITERL.

Figure 2 shows the hardware architecture of ITERL, which is described in detail below. 

### 3.1. Sensing Devices

A set of sensors was used for the measurement of the ambient conditions (illumination, rain, temperature, and humidity), while video cameras were used for vehicle detection. Table 1 shows the selected sensors and the video camera, as well as their main features.

### 3.2. Wireless Nodes

There is a strong demand for wireless solutions in applications where high speed data transmission is not required, but small, cheap, and autonomous (low power) terminals are required, which guarantee secure and reliable communications. Many of these applications require topologies with a large number of nodes and the lowest possible cost per node. The development of IEEE 802.15.4 technology with different protocol stacks has provided a solution for this demand, which has experienced exponential grow in the last decade. IEEE 802.15.4 features low data rates and high energy efficiency, as well as versatility for the deployment of large networks with complex topologies. We would like to mention the existence of protocol stacks such as ZigBee and Rime, as well as some other implementations that allow IP networking, such as micro-IP (uIP) TCP/IP in its V4 and V6 versions. 

**Figure 3:**
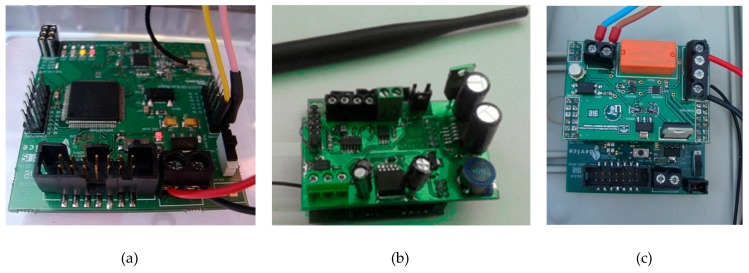
(**a**) Wireless platform hardware; (**b**) sensor node; (**c**) actuator node.

In ITERL, the wireless network collects the data measured by the sensors, establishes communication with the gateway, and receives the data from (transmits the commands to) the streetlight control nodes. The wireless network is composed of three types of nodes: the coordinator node, sensor node, and actuator node. They have a common wireless platform that uses the CC1125 transceiver and the MPS430 family microcontroller, both from Texas Instruments. The MPS430 microcontroller family consists of ultra-low-power devices whose architecture, combined with low power operation modes, is optimized to extend battery life in portable applications. Specifically, the MSP430F5438A device was used here. It integrates general purpose IO pins, a power management stage, and Universal Asynchronous Receiver-Transmitter (UART), Joint Test Action Group (JTAG), and mini USB interfaces. Figure 3a shows the wireless platform hardware developed for the 868 (Europe)/915 (USA) MHz band. 

The hardware of the border router coincides with the wireless platform shown in Figure 3a, since it does not require extra components. This node acts as a coordinator node for the 802.15.4 network. It is in charge of creating the network, managing the routing, and the connection and disconnection of nodes. It receives the messages sent by sensor nodes towards the local controller, and resends the commands received from the local controller to the actuator nodes located in the streetlights. There is only one border router in the network.

For the sensor node (Figure 3b), a specific card was designed to condition the signals coming from sensors. It is plugged into the common wireless hardware platform previously mentioned. It also contains the voltage regulator required to power both the sensors and the sensor node itself. 

**Figure 4:**
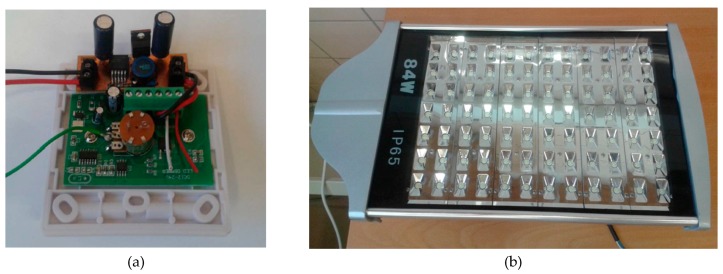
(**a**) Pulse width modulation (PWM) regulator for the control of the streetlights; (**b**) Image of a streetlight.

The actuator node (Figure 3c) controls the on–off function of the streetlights, as well as their illumination level using pulse width modulation (PWM). To this end, a control card was designed that activates and deactivates the streetlights by means of a relay (PE014005), which acts as a power switch. To set the illumination level, from an internal voltage source, a programmable voltage divider built with a digital potentiometer (MAX5160) selects one of 32 different voltage references for the PWM regulator (Figure 4a).

### 3.3. LED Luminaires

An 84 W LED luminaire was selected for ITERL (Figure 4b). It comes with a regulator that raises the voltage level up to 48 V and a dimmer that receives the PWM signal from the voltage regulation card mentioned above. Table 2 shows the technical specifications of the selected streetlight. 

### 3.4. Local Controller, Image Processor, and Gateway

Signals coming from sensors are locally processed in the microcontroller available in their corresponding sensor node. The local controller, built on a BeagleBone Black platform, concentrates the information coming from (and directed towards) the wireless network. Concerning the video cameras, powerful hardware is required to build the image processor, and an industrial computer (the VBOX 3200) was selected for this purpose. The image processor is also in charge of transmitting the data to a remote server using a 3G link, acting as a gateway for ITERL

## 4. Communication Network

### 4.1. Wireless Sensor Network (WSN)

**Figure 5:**
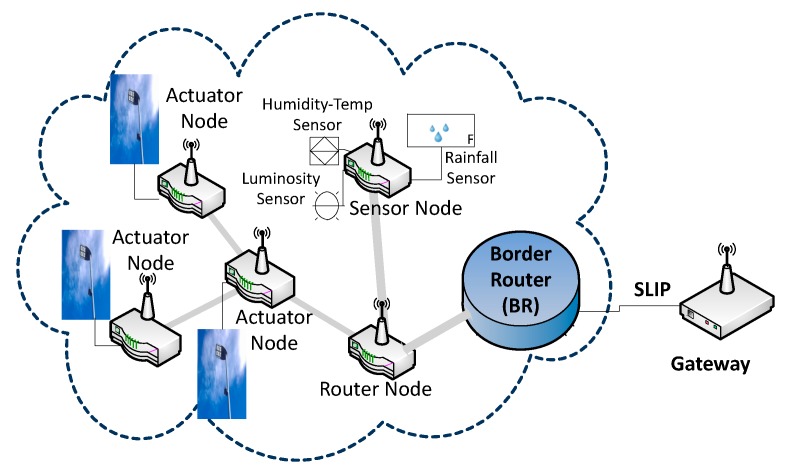
Network topology. Note: SLIP = serial line Internet protocol.

The network topology is shown in Figure 5. It is organized as an IPv6 subnet. At a functional level, the client-server communication paradigm was followed, so that end devices establish User Datagram Protocol (UDP) communication with the coordinator node, which also acts as a 6LowPAN border router (BR). Sensor nodes collect the ambient information (illuminance, ambient temperature, relative humidity, and rain). The coordinator node collects the data coming from sensor nodes and sends them to the communication gateway through a series connection, over which the serial line Internet protocol (SLIP) is implemented. Although a star topology could have been selected, the presence of a router allows that multiple intersections to be controlled in the future by the same local controller. At the actuation level, an actuator node is placed in each of the streetlights to control the illumination level. 

Contiki was chosen as the operating system (OS). Contiki is a small, open source, highly portable, and multi-task-oriented OS developed for small systems, ranging from 8 bit computers to microcontrollers, with an emphasis on wireless sensor networks. Although it has a complete TCP/IP stack, Contiki only requires a few kilobytes of code and several hundred bytes of RAM. 

For data interchange between the Wireless Personal Area Network (WPAN) devices, Contiki incorporates two communication stacks: Rime and uIP. Packets with an IP destination address that matches the address associated with a valid node are registered. If they use the IPv6 protocol, the uIP stack looks for the existence of any extension header and processes it, generating an Internet Control Message Protocol (ICMP) error message in case of errors. The packet is only uploaded to the transport layer when no errors are found when processing the headers, the package length is correct, and the destination address matches the address of the receiving node. The transport protocol can be UDP, TCP, or ICMP (note that although the latter is not a transport protocol, it is considered to be one within the uIP stack). The reduction of program code and uIP memory is obtained by three methods: (**a**) an event-based programming interface, (**b**) a simple memory buffer management scheme, and (**c**) efficient implementation of the TCP protocol.

Several node addresses were used in this work, taking advantage of the different protocol layers offered by Contiki: application address, uIP (network) address, and Rime (link) address. Each of the network devices, at each moment, has one unique address of each type. The protocols chosen for the implementation of each of the layers of the OSI model, as well as the specific configuration parameters of the Contiki OS, were the following:

- Application Protocol: This is in charge of the encoding of the data generated by the sensors. It also defines alarm frames in abnormal situations;
- Transport Protocol: Sensor and actuator nodes establish a UDP connection with the border router. This protocol is preferred to TCP because it is easier to implement;
- Network Protocol: The uIPv6 protocol is chosen. In the Contiki OS, the network driver *sicslowpan_driver* was used, and the *WITH_UIP6 = 1* macro was selected;
- Media Access Control (MAC) Protocol: The Carrier Sense Multiple Access with Collision Avoidance (CSMA/CA) mask is chosen for the medium access control protocol. Therefore, the *csma_driver* was chosen in the Contiki OS;
- RDC Protocol: In the Contiki OS, the driver chosen was the *contikimac_driver*;
- Frame Formation Protocol: Frames are generated in accordance with the IEEE 802.15.4 protocol. In the Contiki OS, the *framer_802154* driver was used;
- Radio Protocol: This defines the transceiver driver. In the Contiki OS, for the CC1125 transceiver, the driver chosen was the *cc1125_driver*.

The wireless network uses a mesh topology with adaptive route optimization. Extensive simulations using COOJA [17], a simulator especially designed for the Contiki OS, was used to optimize throughput. 

### 4.2. Border Router, Local Controller, and Gateway

The border router has the ability to perform bidirectional IPv6 to Ipv4 datagram conversion in order to communicate with the WSN with the web through the gateway. The image processor gateway is responsible for receiving, managing, processing, and storing the network information resulting from communication between the coordinator, sensor and actuator nodes, and the video cameras. In addition, it also processes the commands to act on the streetlights, which are triggered by the arrival of an event.

The communication links between these elements use the following:

- A SLIP interface that links the local controller with the coordinator of the wireless network (i.e., the border router). The use of this protocol allows the transmission of IP packets over a serial line in a simple way. It does not support either error control or frame fragmentation. Contiki uses SLIP to adapt the Ipv6-based wireless network to a higher capacity device, such as the communication gateway through the coordinator node. In the gateway, from the available tools, the *slattach* tool was used, which allows the creation of an interface to connect the coordinator node through a local network address.
- An Ethernet interface that links the local controller with the image processor gateway.
- A UART interface that links the image processor gateway with the 3G modem.

## 5. ITERL Software

In this section, the main functions and algorithms implemented in the devices comprising ITERL are described. 

### 5.1. Event Detection by Cameras

The events to be controlled are related to the arrival of vehicles to some pre-defined areas of interest. Upon detecting these events, the system analyzes them and generates a series of responses in the streetlights. 

Event detection is done using Imageprocess, an application based on the Open source Computer Vision (OpenCV) library for object detection in images. When Imageprocess is active, it takes the real time video obtained from the camera and the pre-defined areas of interest as data sources. When a vehicle enters one of the pre-defined areas of the camera, Imageprocess generates an event that is processed by other programs in the image processor, producing an action on the streetlights. This event contains the ID of the area where the incoming vehicle was detected, the ID of the detected object, the type of detected event, the vehicle direction, the vehicle speed, the size of the detected object, and the date of detection. For each camera, an independent instance of Imageprocess is run, which analyzes its assigned area. 

The camera-based event detection program requires the definition of the so-called areas of interest. They are defined as eXtensible Markup Language (XML) files, which fix their boundaries by means of coordinates in the image plane; a different XML file is required for each area of interest. An Application Programming Interface (API), responsible for analyzing the events generated by image process, was created in Flask, an API microframework in Python language. The first objective of this API is to determine if the received event is relevant. Then, the application checks if it contains the expected information and if it has a valid content. In this case, the API transforms this event into a data frame, which is sent through a TCP socket to the program in charge of managing and storing the system data frames. If the expected information is not present or has invalid content, the event is discarded. 

With respect to movement detection, different algorithms were programmed and tested in the laboratory using a testbench composed of sequences of images taken from academic repositories on the Internet, and from a database of images recorded by ITERL cameras in the demonstration area. This testbench includes images recorded under different conditions (day or night, with good visibility, rain, or fog. 

The algorithm used in ITERL is an evolution of Oriented FAST and Rotated BRIEF (ORB) [18], where the movement is detected by comparing the current photogram with the background, which in turn slowly adapts itself to follow the changes in atmospheric and ambient conditions. The algorithm was tuned in-field to reduce the number of false negatives down to zero under conditions of good visibility, at both day and night. Note that the presence of false negatives under low visibility conditions is not so important, since under these conditions, ITERL is programmed to illuminate the intersection regardless of traffic activity.

### 5.2. Local Controller

**Figure 6:**
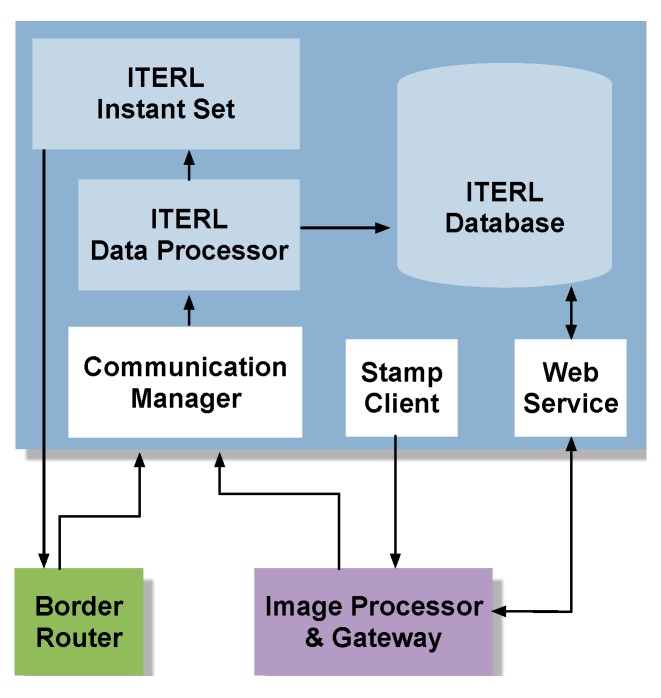
Logical architecture of the local controller.

**Figure 7:**
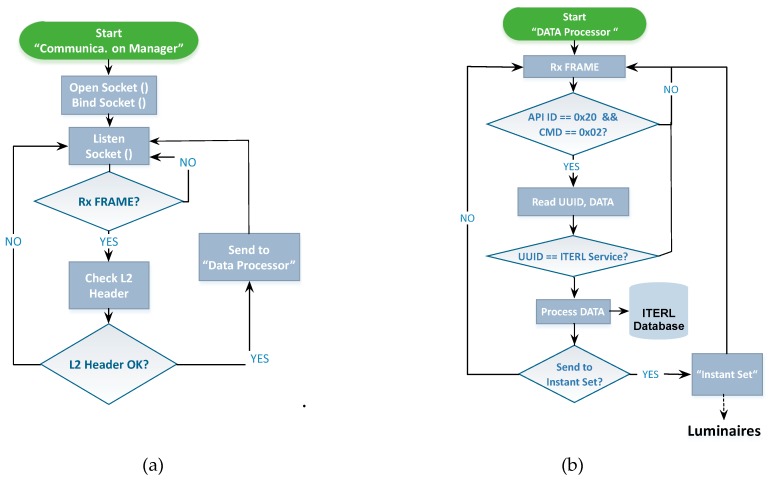
(**a**) Flowchart of the communication manager; (**b**) Flowchart of the data processor.

Figure 6 depicts the logical architecture of the local controller. The communication manager is a server developed in C language that receives information from the wireless network through both the border router and the image processor and sends this information to the internal data processor. The server establishes a communication channel through the creation of a UDP socket. After the socket is created, it is attached to a free listening port and initiates an attention process. It continuously listens to the attached port, and when input data is detected, it starts a checkup function that processes the frame header and checks that the received data (Rx) is correct. If an error is detected in the frame header (incorrect cyclic redundancy check, frame start flag not detected, etc.) it is rejected, and the corresponding port is listened to again. If the header field is correct, then the raw frame is sent to the data processor. Figure 7a shows the flowchart of the communication manager.

As for the data processor in Figure 6, it is responsible for receiving the frames from the communication manager and processing the incoming information. This process follows a state machine, implemented in C++, which goes through the following phases (Figure 7b):

- Reception of one data frame from the communication manager;
- Reading of the frame identification fields:
  ○ First, the API indicator (API ID) and the command type (CMD) are processed to verify that it is a reading-type frame;
  ○ Then, the useful information is stored for later treatment.
- Reading of the service identification Universally Unique IDentifier (UUID) field, with a twofold objective:
  ○ To verify that the service identifier belongs to a sensor measurement or an event; and
  ○ In the case of an event, to take the necessary actions on the streetlights, if required.

The data processor takes different actions depending on whether the information corresponds to a temperature or humidity measurement, to a fog event (requiring the computation of the dew point), to a luminosity measurement, or to an event coming from any of the field cameras. Once a given reading frame is processed and the necessity of actuation on the streetlights is detected, the “instant set” module from Figure 6 is responsible for programming the required action. The actuation frames are sent to the border router in an orderly manner, adding a 0.1 second delay between frames. A re-sending mechanism is implemented to ensure that the actuation frames arrive at the destination.

The “web service” block in Figure 6 is an information request mechanism based on web services, implemented in the local controller for external consultation of system data (i.e., those measurements and events stored in the database). Finally, a method must be provided for time and date synchronization. This is the objective of the “stamp client” module shown in Figure 6. 

### 5.3. Server Database and Graphical Interface

**Figure 8:**
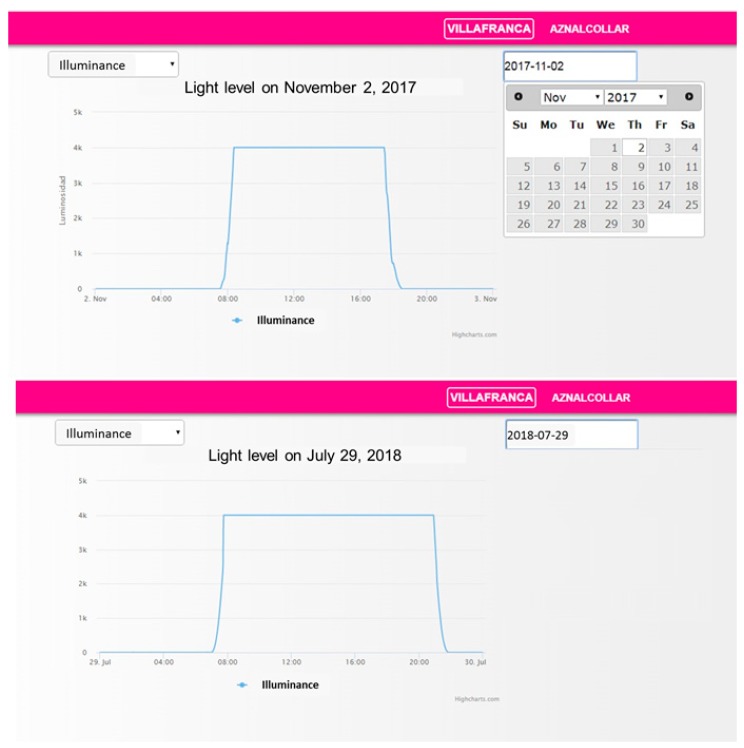
Snapshot of the graphical interface.

A My Structured Query Language (MySQL)-type database was implemented in a remote server, where data coming from cameras and sensors measuring the ambient conditions are stored. This allows the user or analyst to have a historic record of the system’s behavior, as well as a representation of data in a graphic interface. Figure 8 shows snapshots of the graphical interface. It allows the user to choose the measurement to display and the date of consultation (using a calendar). Measurements can be graphically displayed or printed in plain text. Additional pages are available to provide information about the state of ITERL and its components and for system configuration.

It is notable that Figure 8 illustrates the measured ambient illuminance graph for two different dates. Daylight duration can be determined so that the actuation over streetlights is performed when necessary (nighttime, sunrise, and sunset). For the dates in Figure 8:

- On November 2, 2017: From 00:00 to 07:50, and from 17:50 to 24:00.
- On July 29, 2018: From 00:00 to 07:30, and from 21:15 to 24:00.

## 6. Experimental Results

Experimental validation of the ITERL system was carried out using a simplified version consisting of one detection point and four actuation points. The detection points include the sensors that measure ambient conditions and the cameras that detect vehicle presence. The actuation points consist of the LED luminaires (powered by a solar panel) and the actuator node used for their control. Table 3 and Table 4 show the components of the detection and actuation points, respectively, excluding power components (solar panels, regulators, and batteries). 

Regarding the inventory of components in Table 3 and Table 4, the energy consumption was computed and measured in the laboratory. Results are shown in Table 5 (for the detection point) and Table 6 (for each actuation point). The power consumption for the detection and lighting points was 523 and 542 Wh per day, respectively. Two parallel connected solar panels of 100 W and one 12 V, 85Ah battery were selected to supply the detection point. One solar panel of 190 W and two series-connected 12 V, 66 Ah batteries supplied the lighting points, since its power regulator requires a 24 V input voltage.

The correct functioning of the systems was first verified in the laboratory, both for hardware and software components, including the wireless communication network and the energy storage and control subsystem. With respect to the software, the tests consisted of the simulation of a series of events through the communication gateway, followed by an analysis of the system response by comparison to the expected one. Among others, the following tests were performed:

- Detection, reception, and storage of measurements and events, coming from both the wireless network and the cameras;
- Actuation on streetlights after the reception of an event, under different ambient conditions;
- Interactions with the system through the internal web service.

**Figure 9:**
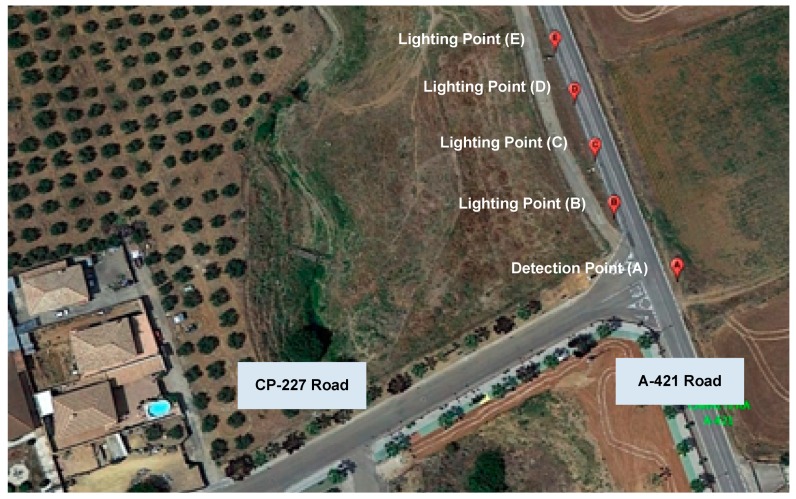
Deployment area for ITERL field test.

Once the tests in the laboratory were completed, the correct operation of the communication manager, the data processor, and the instant set applications was guaranteed, as well as the proper use of the internal database. Only then could field tests be addressed. To this end, a prototype of ITERL was deployed at an intersection in the village of Villafranca de Córdoba, in southern Spain. Figure 9 shows an aerial view of the deployment area, and Table 7 shows the geolocated coordinates of its components.

**Figure 10:**
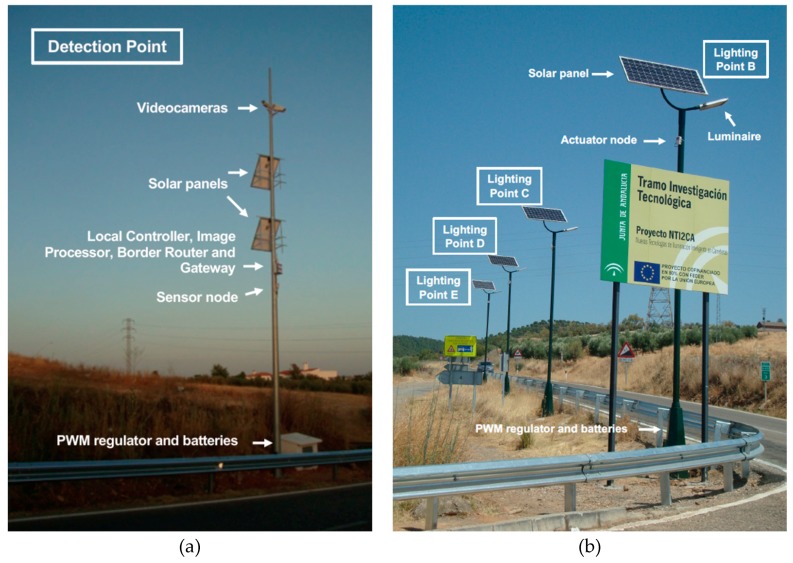
(**a**) Detection point A; (**b**) Lighting points B, C, D, and E.

Following the nomenclature introduced in Figure 2, Figure 10a shows detection point A. Three cameras were placed near the top of a pole at a height of 11 meters, with one of them facing the CP-227 road, and the other two facing both directions of the A-421 road. The cameras covered 200 meters. At a height of 5.5 meters, the sensor node (including the sensors) was placed inside its protection cover. Slightly above the sensor node, at a height of about 6 meters, the image processor and gateway and the border router were placed inside a protective cover. The two solar panels of the detection point, connected in parallel, were placed at heights of 6 and 9 meters, respectively, with a southern orientation and an inclination angle of 40°. The battery and PWM regulator were placed inside a small tamper-proof stand built next to the base of the pole. 

As for the lighting points (B, C, D, and E, respectively), four poles of about 8 meters in height were installed, with a relative separation of approximately 18 meters (Figure 10b). In every pole, a single LED luminaire was used, placed on top of the pole at approximately 8.5 meters high. Point B, the closest to the intersection, was 24 meters away from detection point A. Each lighting point provided enough light for a distance of 20 meters along the road. The actuator node, with its corresponding protective cover, was attached to its corresponding pole near the streetlight. One solar panel, also orientation towards the south and at an inclination angle of 40°, was placed just on top of the pole, at a height of 9 meters. Batteries and power regulators were located inside the bases of each pole, under protective covers. Wiring passed through the interior of the pole. 

Implementation aspects such as the distance between detection, lighting points, and the intersection were selected according to national regulations and industry recommendations [19], under the supervision of experts from our industrial partner organizations and the administration. These values can be considered to be adequate for T-shaped intersections, such as that in Figure 9 with selected streetlights, although they should be carefully reconsidered for each new deployment.

## 7. Discussion

**Figure 11:**
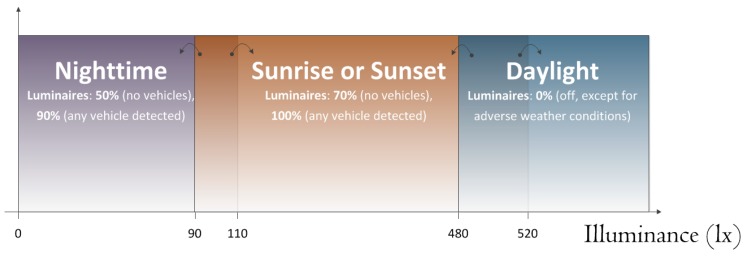
Daytime intervals in ITERL. Illuminance levels correspond to the selected demonstrator.

The ITERL demonstrator was in operation from July 2016 to December 2018. In the first semester, it was under daily supervision from the central facilities, and a report was elaborated by a group of experts from a company in charge of the use and maintenance of traffic infrastructure. As a consequence, the lighting sequence and the intensity level of the streetlights were updated their final version, which is illustrated in Figure 11. According to the average measured illuminance, ITERL split a day into four successive intervals, namely nighttime, sunrise, sunset, and daylight, and a different lighting configuration was assigned to each one. In every interval, LEDs were illuminated at a low lighting level by default and at a high level when a vehicle was detected. Maximum and minimum lighting values were fixed at every interval, although ITERL determined the final value according to the present interval and the instantaneous ambient conditions. As a matter of illustration, by default, LEDs were not active during daylight, even when a vehicle was detected. However, during the daytime, the intersection could be lit to a high level when a vehicle was detected under low visibility conditions (i.e., fog or rain). To accomplish this selection, ITERL had a complete set of decision rules.

Some other general rules were taken into account for the sake of safety, to prevent drivers from being confused or dazzled by sudden changes in lighting:

- A certain hysteresis in the measured illuminance was taken into account when changing from a daytime interval to another one. In addition, the date and the estimated duration of each interval were also taken into account to avoid false changes;
- When a change in the lighting level of a streetlight was decided, it was done in a gradual manner, in 1 second steps of no more than 10% of maximum lighting.

Concerning energy efficiency, Table 8 shows the percentage of power reduction in ITERL with respect to a conventional lighting system, whose streetlights are fully “on” overnight (12 hours), and “off” the rest of the day. It can be seen that the energy consumed by ITERL was about 50% less in many cases, with an average reduction of 36.78%. When compared to the conventional system, ITERL also illuminated the intersection under adverse weather conditions, regardless of the time of day, which is a significant contribution to road safety. 

The contribution of the wireless sensor network to total power consumption was negligible, since even though a wireless node can consume up to 40 mA when transmitting or receiving data, it was in sleep mode most of the time. The highest contributors to total power consumption were the streetlights, although they were seldom illuminated at high values (90% or 100% of their maximum values) due to ITERL intelligence. 

Table 8 also shows the average number of vehicle detection events (excluding the daylight interval) per day for every quarter of the year, which is an indicator of the ITERL activity during the intervals with low illuminance.

## 8. Conclusions

This paper has presented ITERL, an adaptive wireless system for efficient road lighting with an emphasis on rural intersections, which have a high number of road accidents. To achieve efficient power usage, ITERL employs recent LED technology for streetlights and has a smart controller that takes into account the presence and speed of vehicles that approach an intersection as well as ambient conditions in order to illuminate the area of interest with the most appropriate light intensity, only when it is necessary. To this end, ITERL incorporates a set of sensors (illuminance, temperature, humidity, and rainfall) and video cameras (to detect the presence and speed of incoming vehicles). To reduce installation and operation costs, communication between ITERL elements (sensors, actuators, and local controller) is done wirelessly, which does not require civil work. Finally, the local controller is supervised from a central facility, as it is connected using the cellular network provided by a telecommunication carrier. 

The paper provides a detailed description of ITERL operation and of its components. An ITERL prototype was deployed at a road intersection in Villafranca de Córdoba (Spain), where it was in continuous operation for more than two years. Results of ITERL operation and the measured energy savings when compared to a conventional road lighting system were also reported, showing an average power reduction of 36.78%. 

These results show that a smart combination of recent advances in lighting and IoT technologies enable the development of cost-effective road lighting systems, which can make a significant contribution to the reduction of traffic accidents, which is one of the main objectives of transportation departments around the world.

## Figures and Tables

**Table 1:** Selected sensors and main features.

Measured variable	Sensor Reference	Main Features
Illuminance	Trend Control LLO sensor	Selectable detection ranges 4–20 mA interface IP65
Temperature and Humidity	Vaisala HMP110 sensor	0–1 V output range
Rain	Honeywell LLE101000 sensor	Liquid level detection Requires signal adaption
Video Image	Logitech C920 HD camera	Full HD (1920 × 1080 pixels) H.264 video compression

**Table 2:** Main features of the selected streetlight.

General			LEDs
Dimensions	579 × 315 × 204 cm	Correlated Color Temperature (CCT)	6000 K
Chip	EPISTAR Surface-Mount Device (SMD)	Luminous flux	8100 lm
Power	84 W	Angularity	120°
Regulation	1–10 V		

**Table 3:** Summary of components per detection point.

Number	Component	Functionality
**Image Acquisition and Processing**		
3 1	Video cameras Image processor	Vehicle presence and motion detection (speed), including image processing
**Ambient Sensors and Signal Conditioning**		
3	Ambient sensors: Temperature and humidity sensor Light level sensor Rain sensor with respective signal adaption card	Measurement of ambient conditions: Temperature and humidity Illuminance Rain detection, including signal conditioning
**Wireless Sensor Network**		
3	WSN modems	Integration of sensors in the WSN
1	Border router	Border routing, network coordination
**Local Control**		
1	Local controller	Local control
**Remote Connection**		
1	3G modem	ITERL gateway remote connection

**Table 4:** Summary of components per lighting point. Note: WSN = wireless sensor network.

Number	Component	Functionality
**Actuation**		
1	Actuation node	Streetlight control (on–off and dimming)
**Wireless Sensor Network**		
1	WSN modem	Integrating actuators in the sensor network
**Streetlight**		
1	Streetlight	Road lighting

**Table 5:** Estimated consumption of devices used for the detection point.

Consumption Estimation				
Devices	Power (W)	Hours/day	Energy (Wh/day)	Oversizing (Wh/day)
Video cameras	0.25	24	6	7.58
Image processor	18	24	432	545.68
Coordinator node	0.16	24	3.84	4.85
Sensor node and sensors	0.85	24	20.28	25.62
Local controller	1.25	24	30	37.89
Total	21.78		522.72	660.2
**Data of the Installation Implementation Place**				
Peak solar hours (PSH)		4.06		
**Energy Generation**				
Panel peak power		100 W		
Global working factor		90%		
Number of items		660.2/(0.9·4.06·100) = 1.81 ≥ 2		
Maximum panel voltage		18.78 V		
Total number of panels		2 panels of 100 W		
**Storage System**				
Nominal installation voltage		12 V		
Nominal battery voltage		12 V		
Discharge depth		70%		
Required storage capacity		660.2/(0.7·12) = 78.6 Ah		
Number of batteries		1 battery of 12 V and 85 Ah		
**Regulator output current**		1.25·21.78/12 **= 2.27 A**		

**Table 6:** Estimated consumption of devices used for the actuation point.

Consumption Estimation				
Devices	Power (W)	Hours/ day	Energy (Wh/day)	Oversizing (Wh/day)
Actuator Node	0.16	14	2.24	2.83
Pule width modulation (PWM) regulator	0.45	14	6.3	7.96
Streetlights	63	8.5	533	673.26
Total	45.61		541.54	684
**Data of the Implementation Location**				
**Peak solar hours (PSH)**		4.06		
**Energy Generation**				
Peak panel power		190 W		
Global working factor		90%		
Number of Items		684/(0.9·4.06·190) = 0.985 ≥ 1		
Peak panel voltage		36.50 V		
Total number of panels		1 panel at 190 W		
**Storage System**				
Nominal installation voltage		24 V		
Nominal battery voltage		12 V		
Discharge depth		70%		
Required storage capacity		806.6/(0.7·12) = 96 Ah		
Number of batteries		2 batteries at 12 V and 66Ah		
Regulator output current		1.25·45.61/24 = 2.37 A		

**Table 7:** Position coordinates of each element.

Point	Description	Latitude	Longitude
A	Detection Point	37°58’3.88’’ N	4°32’28.07’’ W
B	Lighting Point 1	37°58’4.49’’ N	4°32’28.71’’ W
C	Lighting Point 2	37°58’5.06’’ N	4°32’28.88’’ W
D	Lighting Point 3	37°58’5.63’’ N	4°32’29.08’’ W
E	Lighting Point 4	37°58’6.18’’ N	4°32’29.27’’ W

**Table 8:** Measured consumption improvement per quarter.

	Consumption Reduction of ITERL vs. Conventional Lighting System				
Year	Quarter	Measured Daylight Interval (h)	Average Number of Events (events/day)	Percentage of Assisted Events (%)	Average Reduction of Energy Consumption (%)
2017	Q1	10.08	139	61.87	22.85
	Q2	14.08	108	52.90	48.40
	Q3	14.25	118	55.93	49.87
	Q4	10	122	55.74	24.44
2018	Q1	10.33	135	58.52	29.94
	Q2	13.16	97	53.61	46.14
	Q3	13.25	148	53.89	44.60
	Q4	9.58	116	56.03	28.00
